# Detection of Five *mcr-9*-Carrying *Enterobacterales* Isolates in Four Czech Hospitals

**DOI:** 10.1128/mSphere.01008-20

**Published:** 2020-12-09

**Authors:** Ibrahim Bitar, Costas C. Papagiannitsis, Lucie Kraftova, Katerina Chudejova, Vittoria Mattioni Marchetti, Jaroslav Hrabak

**Affiliations:** a Department of Microbiology, Faculty of Medicine, and University Hospital in Pilsen, Charles University, Pilsen, Czech Republic; b Biomedical Center, Faculty of Medicine, Charles University, Pilsen, Czech Republic; c Department of Microbiology, University Hospital of Larissa, Larissa, Greece; Antimicrobial Development Specialists, LLC

**Keywords:** VIM-4, MCR-9, IncHI2, *Enterobacter cloacae*, *Citrobacter freundii*

## Abstract

The aim of this study was to report the characterization of the first *mcr*-positive *Enterobacterales* isolated from Czech hospitals. In 2019, one Citrobacter freundii and four *Enterobacter* isolates were recovered from Czech hospitals. The production of carbapenemases was examined by a matrix-assisted laser desorption ionization–time of flight mass spectrometry (MALDI-TOF MS) imipenem hydrolysis assay. Additionally, bacteria were screened for the presence of carbapenemase-encoding genes and plasmid-mediated colistin resistance genes by PCR. To define the genetic units carrying *mcr* genes, the genomic DNAs of *mcr*-carrying clinical isolates were sequenced on the PacBio Sequel I platform. Results showed that all isolates carried *bla*_VIM_- and *mcr*-like genes. Analysis of whole-genome sequencing (WGS) data revealed that all isolates carried *mcr-9*-like alleles. Furthermore, the three sequence type 106 (ST106) Enterobacter hormaechei isolates harbored the *bla*_VIM-1_ gene, while the ST764 *E. hormaechei* and ST95 C. freundii included *bla*_VIM-4_. Analysis of plasmid sequences showed that, in all isolates, *mcr-9* was carried on IncHI2 plasmids. Additionally, at least one multidrug resistance (MDR) region was identified in each *mcr-9*-carrying IncHI2 plasmid. The *bla*_VIM-4_ gene was found in the MDR regions of p48880_MCR_VIM and p51929_MCR_VIM. In the three remaining isolates, *bla*_VIM-1_ was localized on plasmids (∼55 kb) exhibiting *repA*-like sequences 99% identical to the respective gene of pKPC-CAV1193. In conclusion, to the best of our knowledge, these 5 isolates were the first *mcr-9*-positive bacteria of clinical origin identified in the Czech Republic. Additionally, the carriage of the *bla*_VIM-1_ on pKPC-CAV1193-like plasmids is described for the first time. Thus, our findings underline the ongoing evolution of mobile elements implicated in the dissemination of clinically important resistance determinants.

**IMPORTANCE** Infections caused by carbapenemase-producing bacteria have led to the revival of polymyxins as the “last-resort” antibiotic. Since 2016, several reports describing the presence of plasmid-mediated colistin resistance genes, *mcr*, in different host species and geographic areas were published. Here, we report the first detection of *Enterobacterales* carrying *mcr*-9-like alleles isolated from Czech hospitals in 2019. Furthermore, the three ST106 Enterobacter hormaechei isolates harbored *bla*_VIM-1_, while the ST764 E. hormaechei and ST95 Citrobacter freundii isolates included *bla*_VIM-4_. Analysis of WGS data showed that, in all isolates, *mcr-9* was carried on IncHI2 plasmids. *bla*_VIM-4_ was found in the MDR regions of IncHI2 plasmids, while *bla*_VIM-1_ was localized on pKPC-CAV1193-like plasmids, described here for the first time. These findings underline the ongoing evolution of mobile elements implicated in dissemination of clinically important resistance determinants. Thus, WGS characterization of MDR bacteria is crucial to unravel the mechanisms involved in dissemination of resistance mechanisms.

## Abstract (precis)

Infections caused by carbapenemase-producing bacteria have led to the revival of polymyxins as the “last-resort” antibiotic. Since 2016, several reports describing the presence of plasmid-mediated colistin resistance genes, *mcr*, in different host species and geographic areas were published.

## OBSERVATION

A significant increase in infections caused by carbapenemase-producing bacteria (1), coupled with the lack of novel antibiotics (2), has led to the revival of polymyxins as the “last-resort” antibiotic (3). Consequently, higher prevalence of colistin resistance among carbapenemase-producing Klebsiella pneumoniae strains has been reported worldwide (4). In K. pneumoniae, resistance to colistin is mainly mediated via chromosomal mutations in genes involved in lipopolysaccharide synthesis (5). However, in 2016, the first plasmid-mediated colistin resistance gene, *mcr-1*, was identified among Chinese Escherichia coli isolates (6). Following the first description, several reports describing the presence of *mcr-1* in different host species and geographic areas were published (7, 8). Thus far, the *mcr* gene family comprises *mcr-1* to *mcr-10* (9). These genes encode phosphoethanolamine transferases that catalyze the addition of phosphoethanolamine to the phosphate group of lipid A, reducing the negative charge of the bacterial outer membrane and attenuating its affinity for colistin, resulting in antibiotic resistance.

Among the *mcr*-like genes, *mcr-1* and *mcr-9* are the most widely disseminated. The *mcr-9* gene has been identified from 40 countries across six continents. However, half of *mcr-9*-positive isolates (1,035/1,682 strains) were recovered in the United States, among which Salmonella enterica was the most common host species, especially in turkeys and chickens (9).

Here, we report the first detection of *mcr-9*-positive members of the *Enterobacterales* isolated from Czech hospitals.

In 2019, 4 isolates belonging to Enterobacter cloacae complex and one isolate belonging to Citrobacter freundii species were recovered from five patients admitted to Czech hospitals (see Table S1 in the supplemental material). In all isolates, which exhibited a meropenem MIC of >0.125 μg/ml (10), carbapenemase production was detected by a positive result in the matrix-assisted laser desorption ionization–time of flight mass spectrometry (MALDI-TOF MS) imipenem hydrolysis assay (11). Screening for carbapenemase-encoding genes by PCR showed that all isolates carried *bla*_VIM_-like genes (12, 13). Additionally, bacteria were positive for the presence of plasmid-mediated colistin resistance genes by PCR, as described previously (14). All VIM-producing isolates exhibited resistance to piperacillin, piperacillin-tazobactam, and cephalosporins, while the variations in the MICs of carbapenems that were observed (Table S2) might reflect the presence of additional resistance mechanisms in some of the isolates. Variations were also observed in the MICs of non-β-lactam antibiotics. However, all isolates were susceptible to colistin, according to data obtained by the broth dilution method (15) and interpreted according to EUCAST criteria (https://www.eucast.org/clinical_breakpoints/).

10.1128/mSphere.01008-20.1 TABLE S1 Susceptibility profiles of *mcr-9*-carrying *Enterobacterales* isolates collected in Czech hospitals during the study. Download Table S1, PDF file, 0.1 MB. Copyright © 2020 Bitar et al. 2020 Bitar et al. This content is distributed under the terms of the Creative Commons Attribution 4.0 International license.

10.1128/mSphere.01008-20.2 TABLE S2 Characteristics of *mcr-9*-carrying *Enterobacterales* isolates recovered from Czech hospitals. Download Table S2, PDF file, 0.1 MB. Copyright © 2020 Bitar et al. 2020 Bitar et al. This content is distributed under the terms of the Creative Commons Attribution 4.0 International license.

To define the genetic units carrying *mcr* genes, the genomic DNAs of *mcr*-carrying clinical isolates were extracted using a NucleoSpin microbial DNA kit (Macherey-Nagel, Düren, Germany) and were sequenced using long-read sequencing technology on the PacBio Sequel I platform (Pacific Biosciences, Menlo Park, CA, USA). Library preparation was done following the manufacturer’s recommendation for microbial multiplexing for the Express kit 2.0 (Pacific Biosciences, Menlo Park, CA, USA). DNA was sheared using Hydropore-long on a Megaruptor 2 device (Diagenode), and no size selection was performed during library preparation. The microbial assembly pipeline offered by SMRT Link v8.0 (Pacific Biosciences) was used to perform genome assembly with a minimum seed coverage of 30. For sequence analysis and annotation, BLAST (www.ncbi.nlm.nih.gov/BLAST), the ISfinder database, and the open reading frame (ORF) finder tool (www.bioinformatics.org/sms/) were used. Comparative genome alignment was performed using Mauve v.2.3.1 (16). Figures were generated from sequence data using BRIG v.0.95 (17).

Analysis of whole-genome sequencing (WGS) data by PubMLST databases (https://pubmlst.org/) revealed that the C. freundii isolate belonged to sequence type 95 (ST95). Additionally, 3 of 4 isolates belonging to E. cloacae complex were ST106, while the remaining isolate was assigned to ST764. *In silico hsp60* typing of the genome sequences showed that four *Enterobacter* isolates belonged to the species Enterobacter hormaechei (18).

Analysis of WGS data using ResFinder 3.2 revealed that all isolates carried plasmid-mediated colistin resistance *mcr-9*-like alleles. Furthermore, the three ST106 *E. hormaechei* isolates harbored the carbapenemase-encoding gene, *bla*_VIM-1_, while the ST764 *E. hormaechei* and ST95 C. freundii isolates included the *bla*_VIM-4_ allele. Also, all isolates included additional genes for resistance to aminoglycosides, tetracyclines, trimethoprim, chloramphenicol, sulfonamides, quinolones, and/or macrolides (Table 1). The presence of the resistance genes was confirmed by the antimicrobial resistance phenotypes (Table S1) of the isolates harboring those genes.

**TABLE 1:** WGS data of *mcr*-carrying *Enterobacterales* isolates recovered from Czech hospitals

Isolate	ST	Replicon of MCR-9- and VIM-encoding plasmids	Plasmid size (bp)	Resistance genes^*a*^	Additional replicons
E. cloacae complex ENCL48212	106	IncHI2 (ST1)	302,551	*mcr*-*9*, *aac*(*6′*)-*IIc*, *aadA2b*, *aph*(6)-*Id*, *dfrA19*, *catA2*, *sul1*, *sul2*, *tetD*, *aac*(*6′*)-*Ib*-*cr*, *qnrA1*, *ere*(*A*), *bla*_SHV-12_, *bla*_TEM-1b_	Col(pHAD28), IncFIB(pECLA)
		pKPC-CAV1193-like	55,220	*qnrS1*, *bla*_TEM-1a_, *bla*_VIM-1_, *sul1*, *aadA2b*, *aac*(*6′*)-*Ib3*	
E. cloacae complex ENCL48946	106	IncHI2 (ST1)	297,470	*mcr*-*9*, *aac*(*6′*)-*IIc*, , *aadA2b*, *aph*(*3″*)-*Ib*, *aph*(6)-*Id*, *dfrA19*, *catA2*, *sul1*, *sul2*, *tetD*, *ere*(*A*), *bla*_TEM-1b_, *bla*_SHV-12_	Col(pHAD28), IncFIB(pECLA)
		pKPC-CAV1193-like	55,222	*aac*(*6′*)-*Ib3*, *aadA2b*, *qnrS1*, *bla*_TEM-1a_, bla_VIM-1__,_ *sul1*	
E. cloacae complex ENCL49790	106	IncHI2 (ST1)	302,836	*mcr*-*9*, *aac*(*6′*)-*IIc*, *aadA2b*, *aph*(*3″*)-*Ib*, *aph*(6)-*Id*, *dfrA19*, *catA2*, *sul1*, *sul2*, *tetD*, *aac*(*6′*)-*Ib*-*cr*, *qnrA1*, *ere*(*A*), *bla*_SHV-12_, *bla*_TEM-1b_	Col(pHAD28), IncFIB(pECLA)
		pKPC-CAV1193-like	55,220	*aac*(*6′*)-*Ib3*, *qnrS1*, *bla*_TEM-1a_, *bla*_VIM-1_, *aadA2b*, *sul1*	
E. cloacae complex ENCL48880	764	IncHI2 (ST1)	262,616	*mcr*-*9.2*, *aac*(*6′*)-*II*, *aadA22*, *dfrA1*, *sul1*, *tetA*, *bla*_VIM-4_	Col(pHAD28), IncFIB(pECLA), IncFII(pECLA), IncR
C. freundii CIFR51929	95	IncHI2 (ST1)/IncM1	369,945	*mcr*-*9*, *aac*(*6′*)-*II*, *aac*(3)-*I*, *aac*(*6′*)-*Ib3*, *ant*(*2″*)-*Ia*, *aadA1*, *aadA2b*, *aph*(*3′*)-*Ia*, *dfrA19*, *catA2*, *cmlA1*, *sul1*, *tetA*, *aac*(*6′*)-*Ib*-*cr*, *qnrA1*, *bla*_VIM-4_	

a *bla*_VIM_- and *mcr*-like genes are underlined.

**FIG 1:**
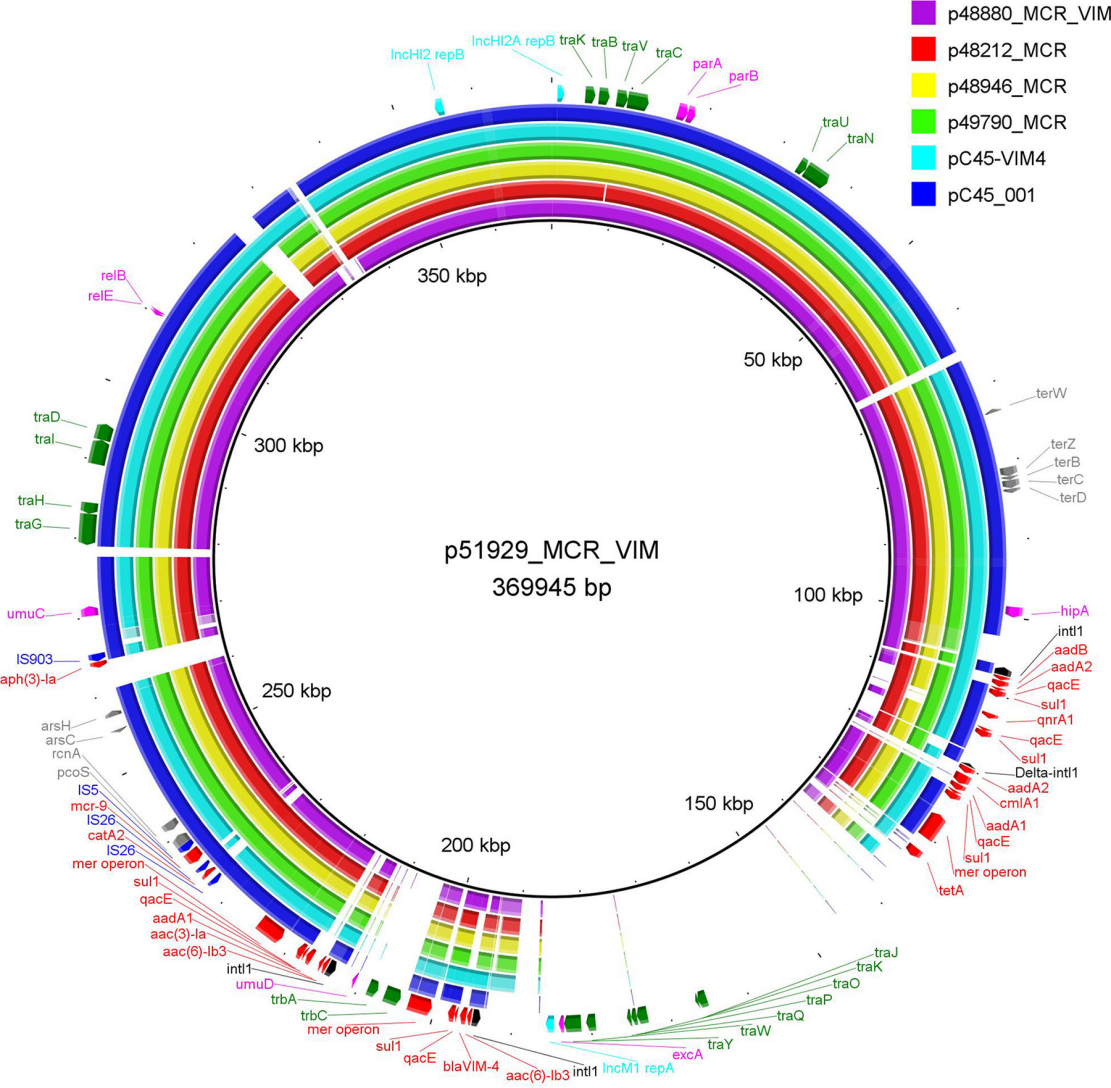
BRIG comparison of IncHI2 *mcr-9*-carrying plasmids characterized from *Enterobacterales* isolates recovered from Czech hospitals.

Analysis of plasmid sequences showed that, in all isolates, the *mcr-9* allele was carried on IncHI2 plasmids (Table 1) (p48212_MCR, p48880_MCR_VIM, p48946_MCR, p49790_MCR, and p51929_MCR_VIM). Plasmids p48212_MCR, p48946_MCR, and p49790_MCR showed high degrees of similarity to each other (99% coverage and 99% identity), while lower diversity was observed in plasmids p48880_MCR_VIM (90% coverage and 99% identity) and p51929_MCR_VIM (77% coverage and 99% identity) compared to p48212_MCR. All plasmids exhibited sequences closely related to other *mcr-9*-carrying IncHI2 plasmids, like pC45-VIM4 from E. cloacae complex isolate C45 (GenBank accession no. LT991958) and pC45-001 from *E. hormaechei* strain C45, recovered from a clinical sample (GenBank accession no. CP042552) in Australia (Fig. 1), and typed as sequence type 1 (ST1) following the IncHI2 pDLST scheme (19). IncHI2 plasmid backbones were composed of regions for replication (*reHI2*), conjugative transfer (*trh* genes), and plasmid maintenance (*par* gene). Additionally, IncHI2 plasmids carried tellurium resistance genes (*terZABCDEF*), commonly associated with this plasmid family, in addition to *terY1*, *terY2*, and *terW* (20). Also, genes conferring arsenic resistance (*arsCBRH*) were found in IncHI2 plasmids. In all IncHI2 plasmids, the *mcr-9* allele was inserted upstream the *pcoS* gene, as observed in other IncHI2 plasmids like pC45-001 (GenBank accession no. CP042552). In all IncHI2 plasmids except p48880_MCR, the *mcr-9* gene was bounded by an IS*903B* element (upstream) and an ORF (downstream), encoding a cupin fold metalloprotein, followed by IS*26*. However, in plasmid p48880_MCR, carrying the *mcr-9.2* allele, an IS*1R* insertion sequence was found downstream of the *mcr-9.2* gene. In all isolates, *qseC* and *qseB* regulatory genes were not found in association with the *mcr-9* gene. Based on previous studies (21, 22), in the presence of subinhibitory concentrations of colistin, *qseC* and *qseB* genes can induce the expression of the *mcr-9* gene, leading to increased MICs. The data mentioned above may explain the susceptibility to colistin.

The *mcr-9* genes from all clinical strains were transferred to the azide-resistant laboratory strain E. coli A15 by conjugation, confirming the ability of IncHI2 plasmids to further disseminate *mcr-9* in other clones or species.

Moreover, at least one multidrug resistance (MDR) region was identified in each *mcr-9*-carrying IncHI2 plasmids. Differences in MDR regions were observed among *mcr-9*-carrying IncHI2 plasmids. Interestingly, the carbapenemase-encoding gene *bla*_VIM-4_ was found in the MDR regions of IncHI2 plasmids p48880_MCR_VIM and p51929_MCR_VIM, as previously described for plasmid pME-1a, which was characterized from an Enterobacter hormaechei isolate harboring *bla*_VIM-4_ and *mcr-9*, recovered from a pediatric patient in a U.S. hospital (21). In plasmid p48880_MCR_VIM, the *bla*_VIM-4_ gene was part of the class 1 integron In416, comprising the *bla*_VIM-4_, *aacA7*, *dfrA1*, Δ*aadA1*, and *smr2* cassettes, while the class 1 integron In1174, which includes an array of *aacA4* and *bla*_VIM-4_ gene cassettes, was identified in plasmid p51929_MCR_VIM.

**FIG 2:**
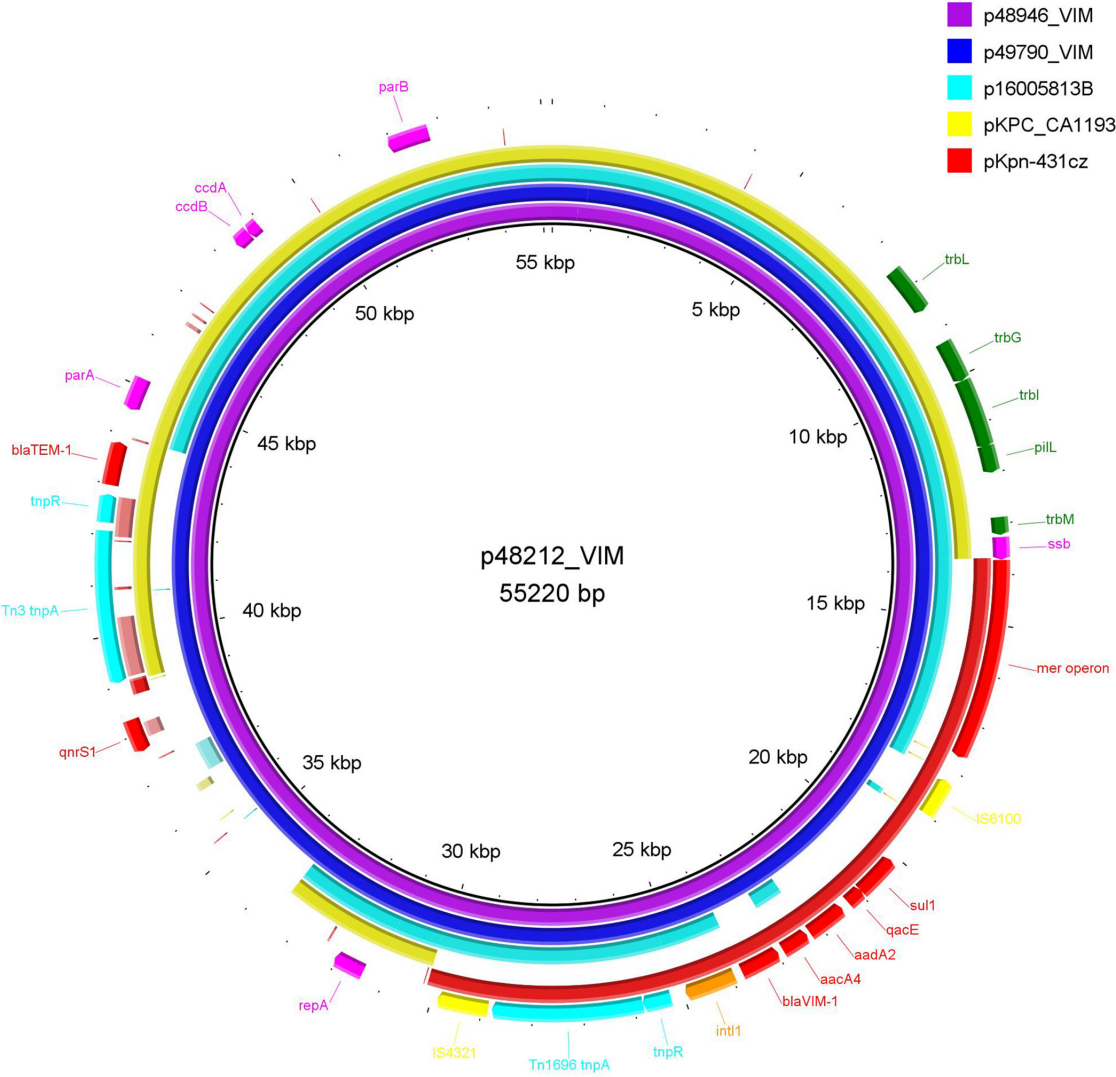
BRIG comparison of VIM-1-encoding pKPC-CAV1193-like plasmids characterized from *Enterobacterales* isolates recovered from Czech hospitals.

On the other hand, in isolates ENCL48212, ENCL48946, and ENCL49790, the *bla*_VIM-1_ gene was localized on plasmids (p48212_VIM, p48946_VIM, and p59790_VIM) of approximately 55 kb. The *bla*_VIM-1_-carrying plasmids shared extensive similarity with plasmid p16005813B from Leclercia adecarboxylata strain 16005813 (72% coverage and 99% identity; GenBank accession no. MK036884) (Fig. 2), encoding IMP-8 carbapenemase. The *bla*_VIM-1_-carrying plasmids could not be typed by the PCR-based replicon typing (PBRT) method (23). However, in the plasmid sequences, *repA*-like sequences of 612 bp exhibiting 99% identity with the *repA* gene of pKPC-CAV1193 (GenBank accession no. CP013325) from Klebsiella pneumoniae strain CAV1193 were identified. Additionally, a complete transfer region was not found in pKPC-CAV1193-like plasmids, explaining the failure of *bla*_VIM-1_-positive plasmids to be transferred, by conjugation experiments, to the azide-resistant laboratory strain E. coli A15, which was used as a recipient.

The MDR regions of VIM-1-encoding plasmids included the class 1 integron In110, whose variable region comprised *bla*_VIM-1_, *aacA4*, and *aadA1* (24). In all three VIM-1-encoding plasmids, In110 was localized in a Tn*1696*-like sequence (nucleotides 13689 to 30156 in p48212_VIM). The IRi of In110 was located between the *resI* and *resII* sites of the Tn*1696* module in precisely the same position as In4 in Tn*1696*. The 3′ conserved segment of the integron was bounded with a Tn*1696* fragment, consisting of IS*6100*, the *resI* site, and the *mer* operon. The Tn*1696*-like transposons were flanked by IRtnp and IRmer of Tn*1696*, with IRtnp being disrupted by IS*4321* while IRmer remained intact. Target site duplications of 6 bp ( CAGCAG) were identified at the boundaries of IRs of the Tn*1696*-like sequence, indicating its transposition within pKPC-CAV1193-like plasmids. Interestingly, resistance islands composed of the class 1 integron In110 associated with a Tn*1696*-like sequence have been previously identified in plasmids pKpn-431cz and pLec-476cz, characterized from VIM-1-producing *Enterobacterales* isolates of Czech origin (25). Additionally, in p48212_VIM, p48946_VIM, and p59790_VIM plasmids, the resistance genes *bla*_TEM-1_, as part of the Tn*3* transposon, and *qnrS1* were found.

In conclusion, to the best of our knowledge, these 5 isolates were the first *mcr-9*-positive bacteria of clinical origin identified in the Czech Republic (Fig. S1). Previous reports from the Czech Republic described the emergence of the *mcr-1.1* allele in *Enterobacterales* recovered from retail meat and the *mcr-4.3* allele in an Acinetobacter baumannii strain isolated from a clinical sample (26, 27). Despite the fact that all 5 *mcr-9*-carrying isolates were colistin susceptible, the identification of these isolates highlights the risk for the hidden spread of important resistance determinants such as plasmid-mediated colistin resistance genes. Additionally, these 5 isolates cocarried the carbapenemase-encoding gene *bla*_VIM_ and several other resistance genes that conferred resistance to aminoglycosides, tetracyclines, trimethoprim, chloramphenicol, sulfonamides, quinolones, and/or macrolides (Table 1), limiting therapeutic choices.

10.1128/mSphere.01008-20.3 FIG S1 Geographic map showing the locations of the hospitals where *mcr*-9-carrying *Enterobacterales* isolates were collected during the study. Download FIG S1, PDF file, 0.2 MB. Copyright © 2020 Bitar et al. 2020 Bitar et al. This content is distributed under the terms of the Creative Commons Attribution 4.0 International license.

Based on epidemiological data, the 5 *mcr-9*-carrying isolates were recovered from three different hospitals, with two of them belonging to the same territory, suggesting three independent insertion events of MCR resistance mechanisms in Czech hospitals. In agreement with epidemiological data, the genomic data confirmed this suggestion. *E. hormaechei* isolates ENCL48212, ENCL48946, and ENCL49790 belonged to the same sequence type (ST106) and harbored similar IncHI2 plasmids carrying *mcr-9.1* and similar pKPC-CAV1193-like plasmids carrying *bla*_VIM-1_. On the other hand, the C. freundii CIFR51929 isolate included an IncHI2 plasmid cocarrying *mcr-9.1* and *bla*_VIM-4_ resistance genes. In plasmid p51929_MCR_VIM, the *bla*_VIM-4_ gene was part of the class 1 integron In1174. Finally, the *E. hormaechei* isolate ENCL48880, which belonged to ST764, harbored the *mcr-9.2* and *bla*_VIM-4_ genes localized on IncHI2 plasmid, p48880_MCR_VIM. In p48880_MCR_VIM, the *mcr-9.2* allele was found in a slightly different genetic environment than the *mcr-9.1* allele in p48212_MCR, p48946_MCR, p49790_MCR, and p51929_MCR_VIM. Unlike p51929_MCR_VIM, the *bla*_VIM-4_ gene was part of the class 1 integron In416 in p48880_MCR_VIM.

The association of the IncHI2 plasmid group with *mcr-1* or *mcr-9* genes has been frequently reported (21, 28). However, the carriage of the *bla*_VIM-1_ gene on pKPC-CAV1193-like plasmids was described for the first time. Interestingly, MDR regions of the *bla*_VIM-1_-carrying plasmids p48212_VIM, p48946_VIM, and p49790_VIM exhibited extensive similarity to the respective regions of plasmid pKpn-431cz (Fig. 2), previously described from *Enterobacterales* recovered from Czech hospitals (25). Thus, the acquisition of the *bla*_VIM-1_-carrying MDR region from pKpn-431cz by a pKPC-CAV1193-like plasmid is a plausible hypothesis regarding the formation of p48212_VIM, p48946_VIM, and p49790_VIM plasmids. These findings highlight the ongoing evolution of mobile elements implicated in the dissemination of clinically important resistance determinants.

### Data availability.

The genomes and plasmids of ENCL48212, ENCL46946, ENCL49790, ENCL48880, and CIFR51929 have been deposited in GenBank under accession no. CP059413 to CP059417, JACEHD010000001 to JACEHD010000006, CP059422 to CP059426, CP059418 to CP059421, and CP059427 to CP059429, respectively.
