# Supplementary material for: Detection of Five mcr-9-Carrying Enterobacterales Isolates in Four Czech Hospitals
Source: mSphere. 2020 Dec 9;5(6):e01008-20. doi: 10.1128/mSphere.01008-20 (PMC7729258; doi:10.1128/mSphere.01008-20)
Supplement: TABLE S1 [file mSphere.01008-20-st001.pdf]

Isolate	MIC (mg/L)															
	**AMP**	**ATM**	**GEN**	**AMK**	**COL**	**CIP**	**TET**	**PIP**	**TZP**	**CTX**	**CAZ**	**MEM**	**ETP**	**TGC**	**NET**	**TOB**
**48 212**	>128	>16	32	<0.5	0.25	0.5	32	128	64	>8	>16	4	>2	0.25	0.5	4
**A15_48 212**	>128	>16	32	<0.5	0.25	0.5	32	128	64	>8	>16	<0.125	>2	0.5	1	8
**48 946**	>128	>16	>32	<0.5	0.25	0.5	32	128	64	>8	>16	4	>2	0.5	1	>8
**A15_48 946**	>128	>16	>32	<0.5	0.25	<0.063	32	16	8	8	>16	<0.125	0.25	0.5	0.5	2
**49 790**	>128	>16	>32	<0.5	<0.125	0.5	>32	128	64	>8	>16	4	>2	0.5	1	4
**A15_49 790**	>128	<0.125	>32	<0.5	0.25	0.25	>32	32	32	>8	>16	<0.125	0.5	0.5	0.5	8
**48 880**	>128	0.25	<0.25	4	0.25	<0.063	32	64	64	>8	>16	0.5	1	0.5	8	4
**A15_48 880**	>128	<0.125	<0.25	1	0.25	<0.063	32	64	64	>8	>16	0.25	0.25	0.25	4	2
**51 929**	>128	4	8	8	0.25	1	>32	128	64	>8	>16	4	0.063	2	>16	>8
**A15_51 929**	>128	<0.125	4	4	0.25	0.125	>32	32	32	>8	16	<0.125	0.25	0.25	>16	8

MIC, minimum inhibitory concentration; AMP, ampicillin; ATM, Aztreonam; GEN, gentamicin; AMK, amikacin; CIP, ciprofloxacin; COL, colistin; TET, tetracycline; PIP, piperacillin; TZP, piperacillin-tazobactam; CTX, cefotaxime; CAZ, ceftazidime; MEM, meropenem; ETP, ertapenem; TGC, tigecycline; NET, netilmicin; TOB, tobramycin.
