# Supplementary material for: Detection of Five mcr-9-Carrying Enterobacterales Isolates in Four Czech Hospitals
Source: mSphere. 2020 Dec 9;5(6):e01008-20. doi: 10.1128/mSphere.01008-20 (PMC7729258; doi:10.1128/mSphere.01008-20)
Supplement: TABLE S2 [file mSphere.01008-20-st002.pdf]

Isolate	ST	Hospital	Specimen	Date of collection	Diagnosis
E. cloacae complex ENCL48212	106	TA	Urine	1/2019	Stroke
E. cloacae complex ENCL48946	106	CB	Urine	2/2019	Multiple Myeloma
E. cloacae complex ENCL49790	106	CB	Decubitus swab	3/2019	Diabetes Mellitus
E. cloacae complex ENCL48880	764	KO	Sputum	1/2019	Pneumonia
C. freundii CIFR51929	95	VFN	Rectal swab	7/2019	Gastric Ulcer
