# Supplementary figures and images for: Detection of Five mcr-9-Carrying Enterobacterales Isolates in Four Czech Hospitals
Source: mSphere. 2020 Dec 9;5(6):e01008-20. doi: 10.1128/mSphere.01008-20 (PMC7729258; doi:10.1128/mSphere.01008-20)

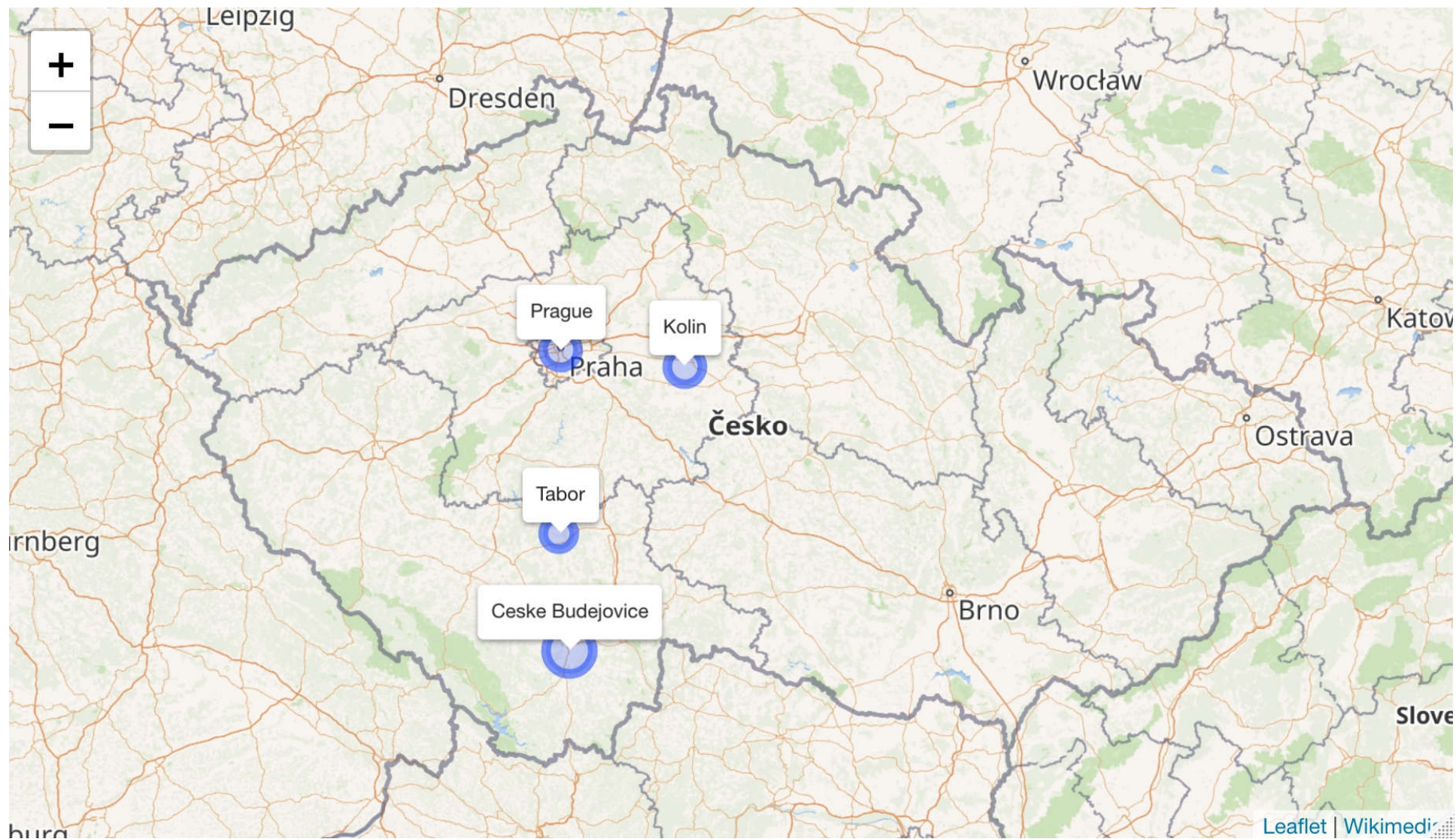

Supplement: FIG S1 [file mSphere.01008-20-sf001.pdf]
